# Dissecting the Thermodynamics of ATP Binding to GroEL One Nucleotide at a Time

**DOI:** 10.1021/acscentsci.2c01065

**Published:** 2023-02-20

**Authors:** Thomas Walker, He Mirabel Sun, Tiffany Gunnels, Vicki Wysocki, Arthur Laganowsky, Hays Rye, David Russell

**Affiliations:** † Department of Chemistry, Texas A&M University, College Station, Texas 77843, United States; ‡ Department of Chemistry and Biochemistry, The Ohio State University, Columbus, Ohio 43210, United States; § Department of Biochemistry & Biophysics, Texas A&M University, College Station, Texas 77843, United States

## Abstract

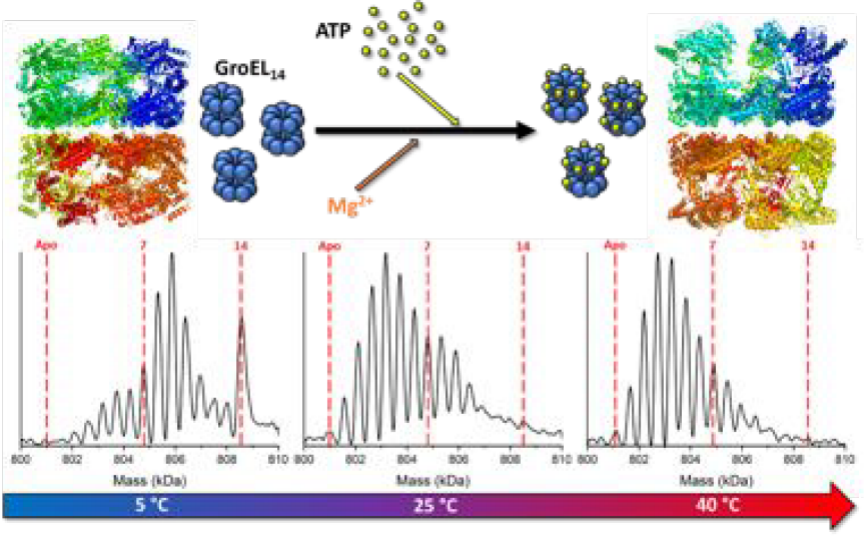

Variable-temperature electrospray ionization (vT-ESI) native mass spectrometry (nMS) is used to determine the thermodynamics for stepwise binding of up to 14 ATP molecules to the 801 kDa GroEL tetradecamer chaperonin complex. Detailed analysis reveals strong enthalpy–entropy compensation (EEC) for the ATP binding events leading to formation of GroEL–ATP_7_ and GroEL–ATP_14_ complexes. The observed variations in EEC and stepwise free energy changes of specific ATP binding are consistent with the well-established nested cooperativity model describing GroEL–ATP interactions, *viz.*, intraring positive cooperativity and inter-ring negative cooperativity (DyachenkoA.; Proc. Natl. Acad. Sci. U.S.A.2013, 110, 7235−72392358987610.1073/pnas.1302395110PMC3645570). Entropy-driven ATP binding is to be expected for ligand-induced conformational changes of the GroEL tetradecamer, though the magnitude of the entropy change suggests that reorganization of GroEL-hydrating water molecules and/or expulsion of water from the GroEL cavity may also play key roles. The capability for determining complete thermodynamic signatures (Δ*G*, Δ*H*, and −*T*Δ*S*) for individual ligand binding reactions for the large, nearly megadalton GroEL complex expands our fundamental view of chaperonin functional chemistry. Moreover, this work and related studies of protein–ligand interactions illustrate important new capabilities of vT-ESI-nMS for thermodynamic studies of protein interactions with ligands and other molecules such as proteins and drugs.

## Abstract 2

A novel native mass spectrometry method for determining the thermodynamics of ligand binding to protein complexes, *viz.*, enthalpy−entropy compensation, is applied to the binding of ATP and ADP to the chaperonin GroEL.

## Introduction

Understanding how large, oligomeric protein complexes respond to the binding of small ligands and other proteins is essential for describing the molecular basis of life. This in turn requires a complete characterization of the binding energetics and correlation of thermodynamic data with interacting structures, including effects of small molecules and solvent. However, the size of many protein oligomers, the myriad intermediate ligation states they can populate, and their often complex allosteric regulation typically restrict analysis by traditional methods to low-resolution ensemble averages. Electrospray ionization native mass spectrometry (ESI-nMS) has evolved as a powerful method for studies of protein–cofactor, protein–ligand, and non-covalent protein–protein interactions. MS-based strategies are attractive because they require only very small amounts of sample, permit product stoichiometries to be directly obtained, and allow reaction kinetics and thermodynamics to be quantified. Klassen and co-workers recently reported a new strategy, “quantifying biomolecular interactions using slow mixing mode (SLOMO) novel nanoflow ESI-MS”, for determination of equilibrium binding affinities (*K*_D_ at 25 °C) for biomolecular interactions,^1^ and they demonstrated the utility of this approach for a number of peptide– and protein–ligand systems. Interactions of proteins with cofactors, ligands, and other proteins often result in conformational changes and/or reorganization of solvent that result in substantial shifts in enthalpy (Δ*H*) and entropy (−*T*Δ*S*). At the same time, solution temperature, pressure, and concentration can influence changes in the “native” structure of biomolecules through manipulation of these thermodynamic contributions.^2,3^ Changes in thermodynamic contributions are often hallmarks of changing conformational states and vice-versa.^3^ Thermodynamic analysis is thus a powerful approach for probing fundamental mechanisms as well as for extracting critical information on structure–activity relationships that is important for drug discovery and drug design,^4^ including water-mediated interactions.^5^

Electrospray ionization produces ions by formation of nanodroplets from which solvent rapidly evaporates, cooling the nanodroplet contents to temperatures of 130–150 K.^6^ Beauchamp and co-workers described this evaporative drying process as “freeze-drying”.^6^ We have used this approach to track the structural evolution of hydrated biomolecules en route to forming solvent-free gas-phase ions,^7^ and we as well as others have shown that this approach can be used to capture native and non-native protein states that coexist in solution. There exist important parallels between cryogenic electron microscopy (cryo-EM) ESI-MS and cryogenic ion mobility (cryo-IM)-MS (*vide infra*) in that both techniques take advantage of kinetic trapping of molecules as they exist in solution.^8,9^

While traditional solution-based techniques can be used to robustly examine temperature-dependent interactions between biomolecules and their ligands, they generally report on the ensemble average of ligand-bound states present in solution. Recent work leveraging the molecular resolution of nMS has shown that species-resolved thermodynamic analysis is possible.^10−12^ Combined with nMS, variable-temperature ESI (vT-ESI) allows for thermodynamic measurements of solution-phase structures with the benefit of mass separation.^11,13,14^ This type of species-specific thermodynamic analysis can be especially valuable for complex or heterogeneous protein–ligand systems where the binding mechanism fundamentally changes as a result of a perturbation or shift in conditions without a measurable alteration to the observed Gibbs free energy (Δ*G*). In these cases, enthalpic and entropic contributions to the Gibbs free energy shift in opposite directions, a phenomenon known as enthalpy–entropy compensation (EEC).^15,16^

We previously reported EEC results for protein complex–lipid binding that varied with lipid headgroup and tail length and showed that mutant forms of AmtB that altered the phosphatidylglycerol (PG) binding site resulted in distinct changes in the thermodynamic signatures for binding PG.^10^ VT-nESI studies of lipid binding to the human G-protein-gated inward rectifier potassium channel, Kir3.2, display distinct thermodynamic strategies to engage phosphatidylinositol (PI) and phosphorylated forms thereof. The addition of a 4′-phosphate to PI results in an increase in favorable entropy. PI with two or more phosphates exhibits more complex binding, where lipids appear to bind to nonidentical sides on Kir3.2; interactions of 4,5-bisphosphate-PI (PI(4,5)P_2_) with Kir3.2 is solely driven by large, favorable entropy, whereas adding a 3′-phosphate to PI(4,5)P_2_ displays altered thermodynamics.^11^ The lipid acyl chain has a marked impact on binding thermodynamics and in some cases results in favorable enthalpy. More recent studies using vT-ESI-nMS combined with ion mobility showed that the cysteine desulfurase enzyme IscU exists in structured, intermediate, and disordered forms that rearrange to more extended conformations at higher temperatures. Comparison of Zn-IscU and apo-IscU reveals that Zn(II) binding attenuates the cold/heat denaturation of IscU, promotes refolding of IscU, favors the structured and intermediate conformations, and inhibits formation of the disordered high-charge states.^17^ Collectively, these studies highlight how vT-ESI-nMS can be applied as a powerful approach for studies of relationships among temperature, conformation, and ligand interactions in a complex biomolecular system.

Molecular chaperones represent another large class of essential biomolecules for which ligand binding and conformational changes are intimately linked to function. The chaperonin family of molecular chaperones, or Hsp60s, are large oligomeric protein complexes that utilize the energy of ATP hydrolysis to actively facilitate protein folding. The canonical bacterial chaperonin, GroEL, is an 801 kDa tetradecamer protein complex from *Escherichia coli* that consists of two heptameric stacked rings. Each GroEL subunit consists of three domains: apical, intermediate, and equatorial.^18−20^ The apical domain is highly dynamic and is responsible for binding protein substrates and the co-chaperonin GroES. The intermediate domain acts as a hinge between the equatorial and apical regions of each subunit, and the equatorial domain of each subunit is the least dynamic and serves as the interfacial contact with the other heptameric ring.^21,22^ The equatorial domain also harbors the ATP binding site for each subunit.^19^ While the structure, dynamics, and ATP binding of GroEL have been extensively investigated,^23−32^ a number of fundamental questions about how ATP binding and hydrolysis control the GroEL functional cycle remain unresolved.

A particular limitation for addressing many of the fundamental issues is the complexity of the GroEL–ATP system, *i.e.*, the tetradecameric complex can bind up to 14 nucleotides. At the same time, ATP binding to the GroEL tetradecamer is known to induce changes in the tertiary structure of the GroEL subunits while simultaneously rearranging the quaternary structure of the oligomer.^27,33^ It has been shown *via* X-ray crystallography and cryo-EM that the binding of ATP by GroEL induces an extension and twisting of the apical domain^32^ and a small “rocking” of the equatorial domain.^33^ The binding of ATP by GroEL is also influenced by the presence of cations (*e.g.*, Mg^2+^ and K^+^): Mg^2+^ is necessary for the binding of ATP, and K^+^ is thought to activate the ATPase activity of GroEL.^34^ It has also been proposed that NH_4_^+^ ions can act as a surrogate for K^+^ ions.^35−37^ These nucleotide-driven structural rearrangements interact to create a complex and layered set of allosteric transitions that govern the GroEL protein folding cycle. This ligand binding and structural complexity constrains the details that can be confidentially extracted from ensemble thermodynamic studies. Dyachenko *et al.* showed that ethylenediammonium diacetate (EDDA) solutions increased the accuracy of mass measurement for GroEL by reducing charge states and minimizing the association of water or buffer molecules,^38^ thereby providing cleaner mass spectra from which the distribution of ligand-bound states could be resolved.

Recently, we reported results using nMS that reveal new insights about GroEL oligomer stability and the stoichiometry of GroEL–ATP/GroES interactions.^39^ Using vT-ESI, we found that GroEL–ATP binding was temperature- and ATP-concentration-dependent, suggesting that more detailed thermodynamic analysis might reveal new insights into how the GroEL nanomachine operates. Here we report thermodynamic measurements (Δ*H*, ΔS, and Δ*G*) for ATP and ADP binding to GroEL utilizing nMS with vT-ESI, along with studies that observe how endogenous ions affect the GroEL–ATP binding interaction. We demonstrate that this approach can be used to dissect the thermodynamic measurements for binding one nucleotide at a time. We further show that EEC plays a central role in the mechanism for binding of ATP to GroEL that may also govern the mechanisms of GroEL functions. These results thus lay the groundwork for the development of new strategies for detailed thermodynamic analysis of other complex, multidentate biomolecular systems. The ability to measure and understand the underlying thermodynamics of protein–cofactor, protein–ligand, and protein–protein interactions has been the focus of much research, particularly in drug discovery efforts.^40,41^

## Results and Discussion

### Thermodynamics of GroEL–ATP Binding in Ethylenediammonium Diacetate Buffer

**Figure 1:**
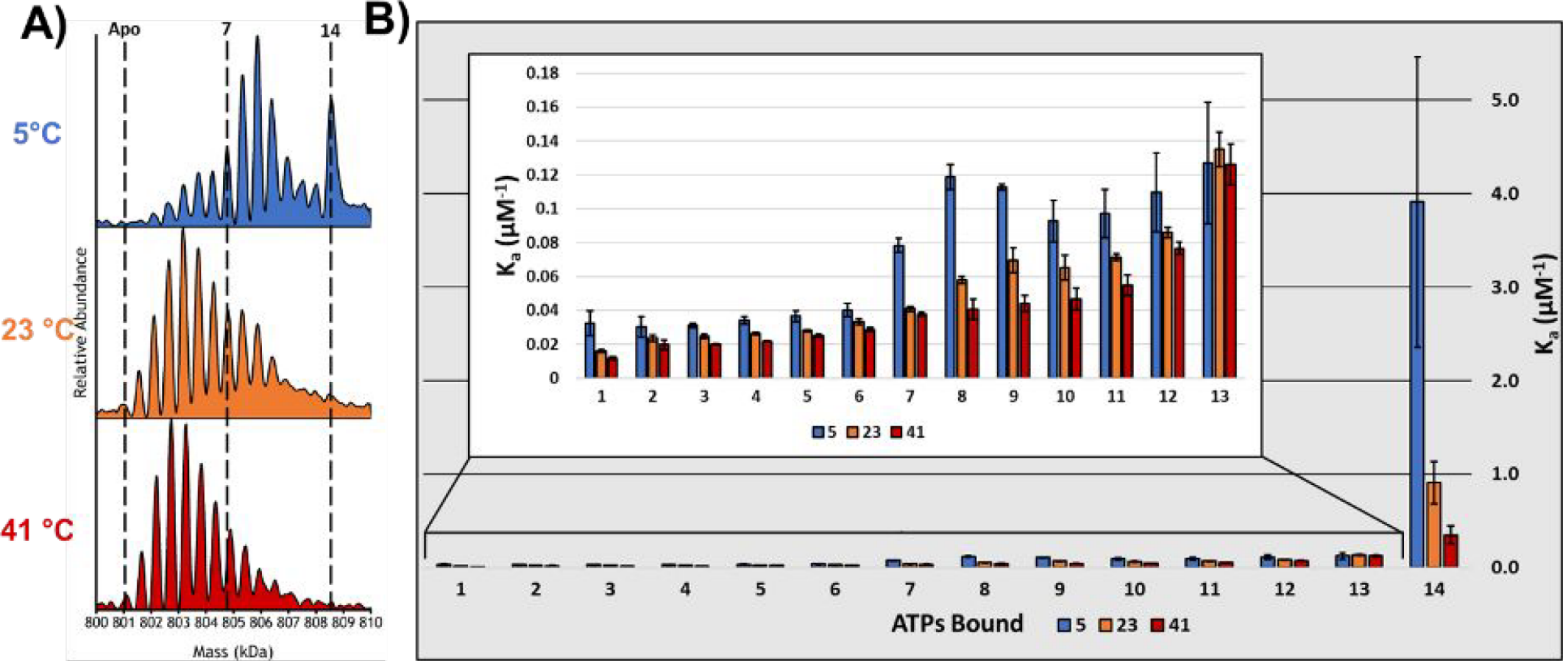
(A) Deconvoluted mass spectra of 500 nM GroEL in a solution of 200 mM EDDA, 1 mM MgAc_2_, and 25 μM ATP at three different temperatures (5, 23, and 41 °C). The binding affinity diminishes as the solution temperature is increased. Bimodality in the ATP binding distributions is likely to be a consequence of negative cooperativity between the *cis* and *trans* rings. (B) Bar chart showing the intrinsic *K*_a_ values calculated for the 14 ATP binding reactions for GroEL at the three different temperatures. The inset shows a bar chart that expands the first 13 binding reactions so that details may be observed more easily. The binding affinity is temperature-dependent, and lower solution temperatures enhance the effect of inter-ring negative cooperativity, as the affinity for ATP decreases more substantially when binding in the *trans* ring begins.

Figure 1A contains deconvoluted mass spectra obtained for solutions of GroEL containing magnesium acetate (MgAc_2_) and EDDA taken at temperatures of 5, 23, and 41 °C; the mass assignment data for the spectra are shown in Table S1, and temperature-dependent mole fraction plots are shown in Figure S1. Figure 1B contains intrinsic equilibrium constants (*K*_a_) for each GroEL–ATP binding reaction. The intrinsic binding constants are statistically corrected to account for the number of modes in which ligands may associate or dissociate from the complex based on the distribution of available monomers (see Methods).^42^ It is interesting to note that the binding of 14 ATPs to GroEL is only observed at lower temperatures in solutions of 25 μM ATP and that the binding affinity is decreased at higher temperatures. Of equal interest are the changes in the observed abundances and corresponding *K*_a_ values for GroEL–ATP products at 5 °C compared with 23 °C for GroEL–ATP_3–8_ and GroEL–ATP_8–10_ (see Figure S1). Most pronounced among all the *K*_a_ values is that for GroEL–ATP_14_, where the effect of the temperature dependence is most easily observed. A similar effect for the GroEL–ATP_14_*K*_a_ value was reported by Dyachenko *et al.*, although their study was conducted at room temperature.^38^ It is also interesting to speculate that the changes in *K*_a_ for GroEL–ATP_8–11_ may constitute a newly identified, temperature-dependent signature for negative inter-ring cooperativity as originally described by Yifrach and Horovitz.^43^

**Figure 2:**
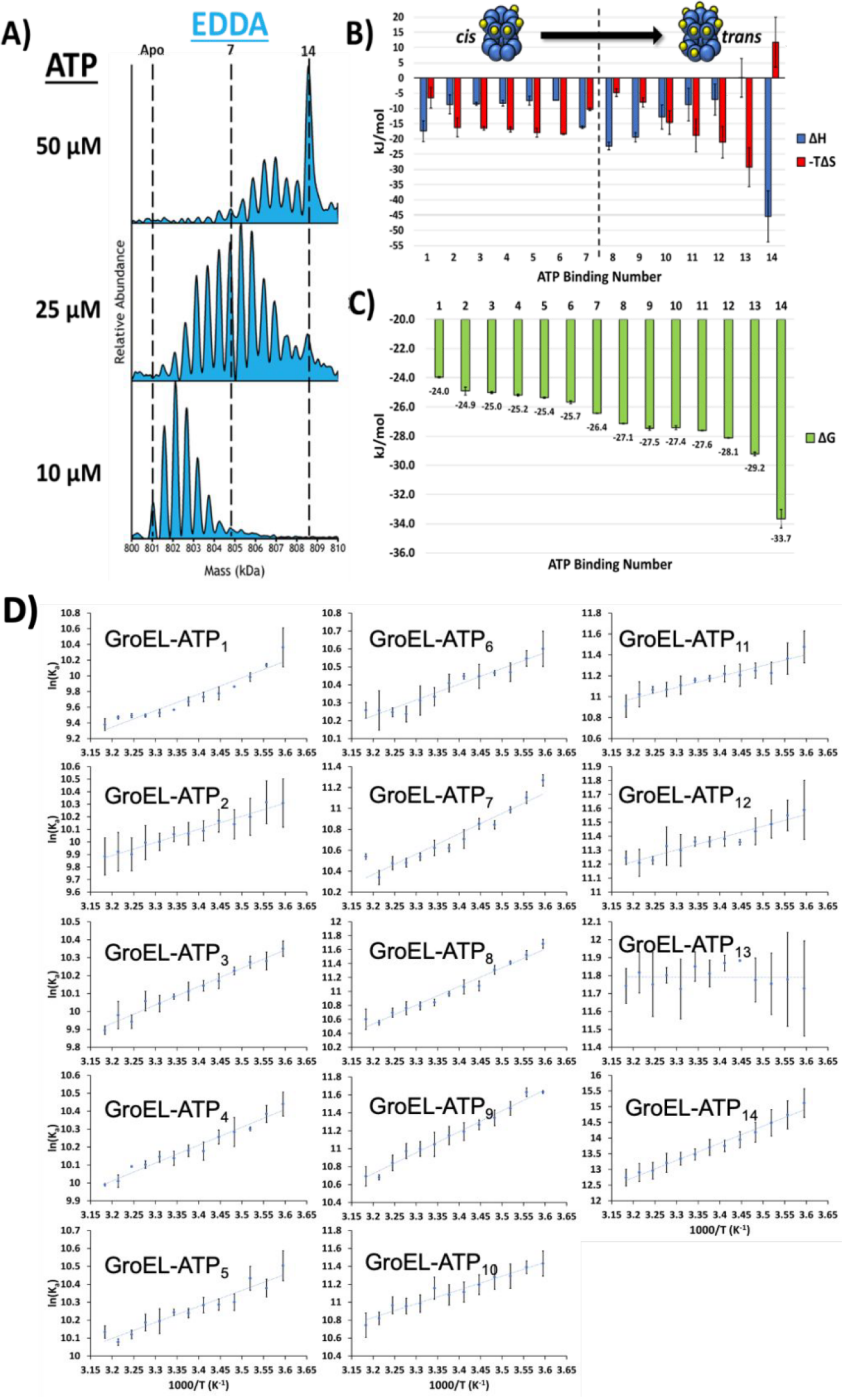
(A) Deconvoluted spectra showing the effects of increasing ATP concentration. (B) Histograms showing enthalpies (Δ*H*) and entropies (−*T*Δ*S*) for the individual ATP binding reactions at 25 °C. The observed EEC for the first seven binding reactions is quite different from that for binding of eight to 14 ATPs, which is consistent with filling of the *cis* ring prior to binding to the *trans* ring. Note also that EEC is drastically different for addition of the 13th and 14th ATP cofactors. These observed changes in EEC are indicative of substantial structural changes in the GroEL complex. (C) Overall changes in Δ*G* associated with the 14 ATP binding reactions. All error bars are standard deviations of three replicates. (D) Van’t Hoff plots (ln *K*_a_*vs* 1000/*T*) for GroEL–ATP_*n*_ binding in 200 mM EDDA.

Figure 2A contains deconvoluted MS spectra for concentration-dependent ATP binding at 25 °C for 10, 25, and 50 μM ATP in EDDA and the absence of magnesium, which prevents GroEL from turning over ATP. These results are consistent with those reported by Cliff *et al.*, who found that low concentrations of ATP promote binding to the *cis* ring to form GroEL–ATP_7_, whereas higher concentrations promote ATP binding to both rings to form GroEL–ATP_14_.^44^ The bimodal binding patterns observed at 23 °C (Figure 1A) and also for solutions containing 25 μM ATP (Figure 2A) may be a result of rearrangement reactions they attributed to structural transitions preceding ATP hydrolysis. Van’t Hoff analysis of the data shown in Figure 1B was used to evaluate the thermodynamics (Δ*G*, Δ*H*, and −*T*Δ*S* at 25 °C) for each ATP binding reaction, and the results are plotted in Figure 2B,C. The van’t Hoff plots for each of the 14 ATP binding reactions are shown in Figure 2D. Interestingly, all of the van’t Hoff plots exhibit a high degree of linearity, which is indicative of no measurable change in heat capacity of the GroEL complex. The ratios for Δ*H* and −*T*Δ*S* illustrate a high degree of EEC, which reflects entropy-driven ATP binding. At low temperature (5 °C), the Δ*G* values show the most diverse pattern for binding of ATP, which would be expected for inter-ring negative cooperativity. Δ*G* values (Figure 2B) for binding of one to seven ATPs range from −24.0 to −26.4 kJ/mol and are even more favorable (−27.1 to −29.2 kJ/mol) for binding eight to 13 ATPs. These observed changes in Δ*G* are consistent with the Hill coefficients reported by Dyachenko *et al.*(38) As the solution temperature increases, these variations in Δ*G* diminish (Figure S2), revealing that binding to the *cis* ring (GroEL–ATP_1–7_) becomes more favored as the temperature is increased compared to that for GroEL–ATP_8–10_.

Enthalpy–entropy compensation, defined as the variation of Δ*H* and *T*Δ*S* in opposite directions, is a strong indicator for conformational changes associated with binding of ATP to GroEL (see Figure 2B,C).^16^ The conformational changes may correspond to changes of the GroEL complex and the water associated with the complex, both water molecules of hydration and those confined in the GroEL cavity;^5,45^ however, the enthalpy term is also subject to similar contributions of structure and solvent.^46^ For example, in the GroEL system, binding of ATP has been attributed to extension of the apical domain and release of confined water, both of which are entropically favorable.^32,47,48^ However, a more extended, labile, and flexible apical domain obviates a loss of favorable (ordered) binding contacts (van der Waals, hydrogen bonding, and salt bridging) that would be enthalpically less favorable. The resultant Δ*H* value for that interaction would be increased and compensated by a decrease in −*T*Δ*S*, *i.e.*, becomes more favorable. Changes in the structure of the GroEL complex are expected to alter the hydration of the complex as well as confined water in the GroEL cavity. A similar change in confined water was reported by Brown and co-workers for transitions between open and closed rhodopsin channel.^48^ Csermely described similar effects of confined water in the GroEL cavity and/or efflux of confined water, both of which are expected to be entropically favored and would be consistent with the EEC trends shown in Figure 2B.^49^

EEC observed for ATP binding rationalizes the modest Δ*G* fluctuations across the range of ATP binding reactions. With the exception of the first and last ATP binding reactions, binding of two to six ATPs to GroEL is largely entropically driven and indicative of structural rearrangement. The observed deviations for EEC in the region of GroEL–ATP_7–10_ coincides with the filling of the first ring and the transition to binding in the second ring. These values switch again for GroEL–ATP_11–13_ and yet again for GroEL–ATP_14_. The binding of the 14th ATP is the only binding reaction with small unfavorable entropy. This seems to indicate that the final structure of the GroEL–ATP_14_ complex is highly ordered compared to preceding binding reactions (GroEL–ATP_11–13_) and is very favored enthalpically with a highly favorable Δ*G* value.

### Effects of NH_4_^+^ Ions on GroEL–ATP Binding

**Figure 3:**
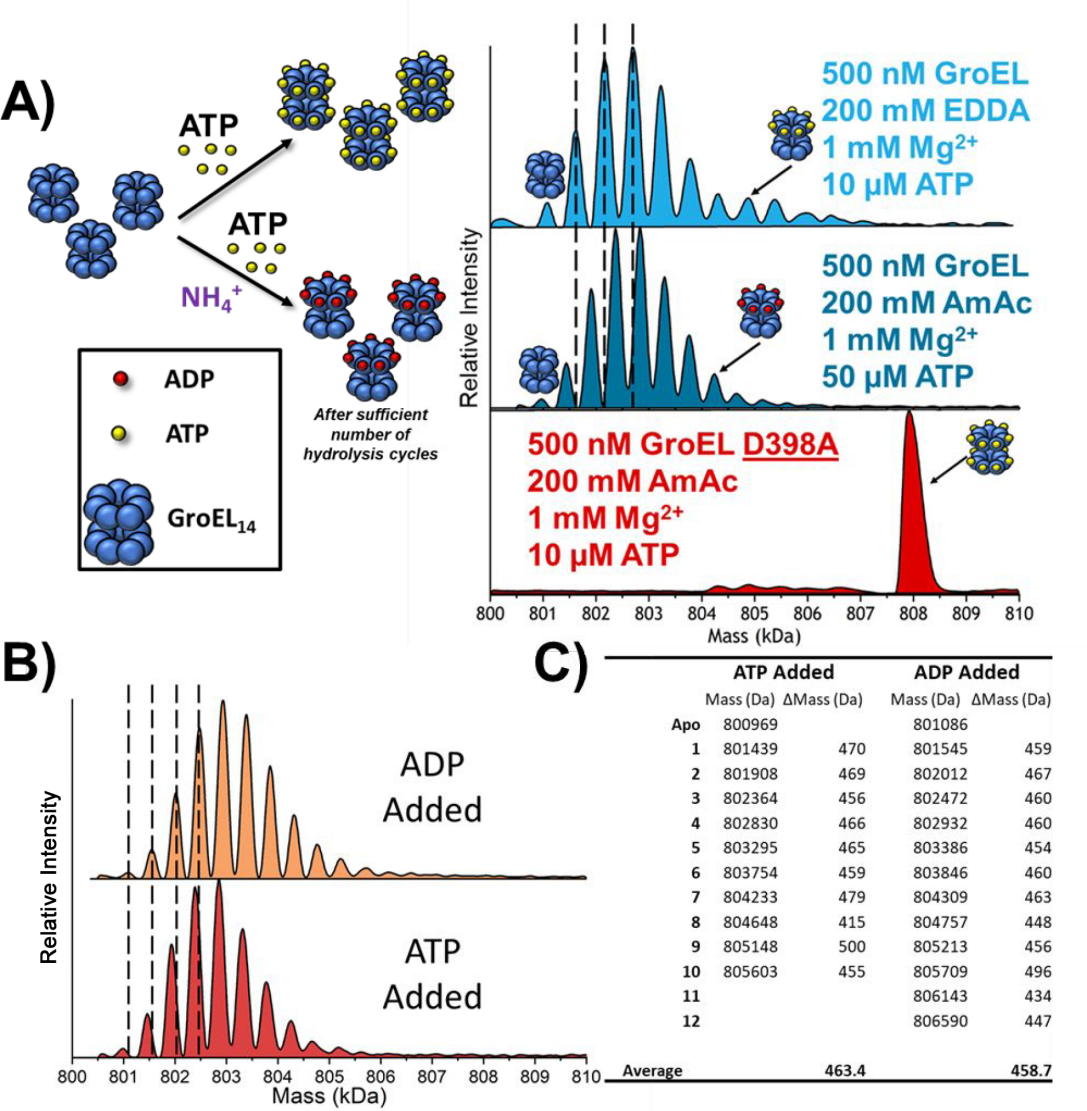
(A) Stacked spectra showing the difference in mass shifts for nucleotide binding observed in EDDA and AmAc solutions. Black lines are used to aid the viewer and show that the mass shifts for EDDA are assigned to [ATP + Mg^2+^]_*n*_ while the mass shifts for AmAc conditions are assigned to [ADP + Mg^2+^]_*n*_. A hydrolysis-deficient mutant GroEL^D398A^ was also analyzed in an AmAc solution (red), which shows the elevated level of cooperative binding in the presence of NH_4_^+^ ions and the absence of hydrolysis. Also note for GroEL^D398A^ that the affinity and cooperativity of the GroEL mutant for ATP are drastically increased in AmAc compared to EDDA conditions. (B) Stacked deconvoluted mass spectra showing the similarities in the binding distributions when either ADP is directly added to a solution containing GroEL or ATP is added under conditions where hydrolysis occurs. Solution conditions are 500 nM GroEL, 50 μM ATP or ADP, 1 mM MgAc_2_, and 200 mM AmAc at 25 °C. (C) Table containing the peak centroid data for (A). Mass shift values are in the form ΔMass = (*n* + 1) – *n*. It should be noted that the measured mass of the apo complex for ATP *vs* ADP is shifted by about 120 Da, explaining why subsequent peaks are not exactly aligned in (B).

In previous work we showed that temperature and ammonium acetate (AmAc) solutions have strong effects on ATP binding, binding with the co-chaperone GroES, and the overall stability of the GroEL tetradecamer.^39^ Lorimer and co-workers showed that K^+^ ions are necessary for the activation of the ATPase mechanism of GroEL.^34,50−52^ Seidel^53^ also reported evidence that NH_4_^+^ ions can act as K^+^ surrogates, which suggests that the observed effects of the ESI buffers may be linked to the initiation of ATPase activity in GroEL. To better understand the effects of NH_4_^+^ ions, we investigated the thermodynamics of GroEL–ATP interactions using two solutions, one containing GroEL in AmAc and the other GroEL in EDDA. The deconvoluted mass spectra in Figure 3A show that signals for nucleotide binding are mass-shifted by ∼80 Da in AmAc compared to EDDA. The observed mass shifts in AmAc solution (mass shifts of 460 Da correspond to [Mg^2+^ + ADP]) are clear evidence that NH_4_^+^ induces GroEL ATPase activity (Figure 3A). We also compared the effects of AmAc on hydrolysis by collecting data for the hydrolysis-deficient GroEL D398A mutant (GroEL^D398A^), and we only observe [Mg^2+^ + ATP] bound to the complex (Figure 3A). It is interesting to note that for GroEL^D398A^, the presence of NH_4_^+^ ions greatly increases the ATP binding affinity. Lastly, we also compared solutions of GroEL in AmAc that contained ADP or ATP, and for both solutions the detected products corresponded to ADP-bound complexes (Figure 3B,C).

### Thermodynamics of GroEL–ATP/ADP Binding in Ammonium Acetate Buffer

**Figure 4:**
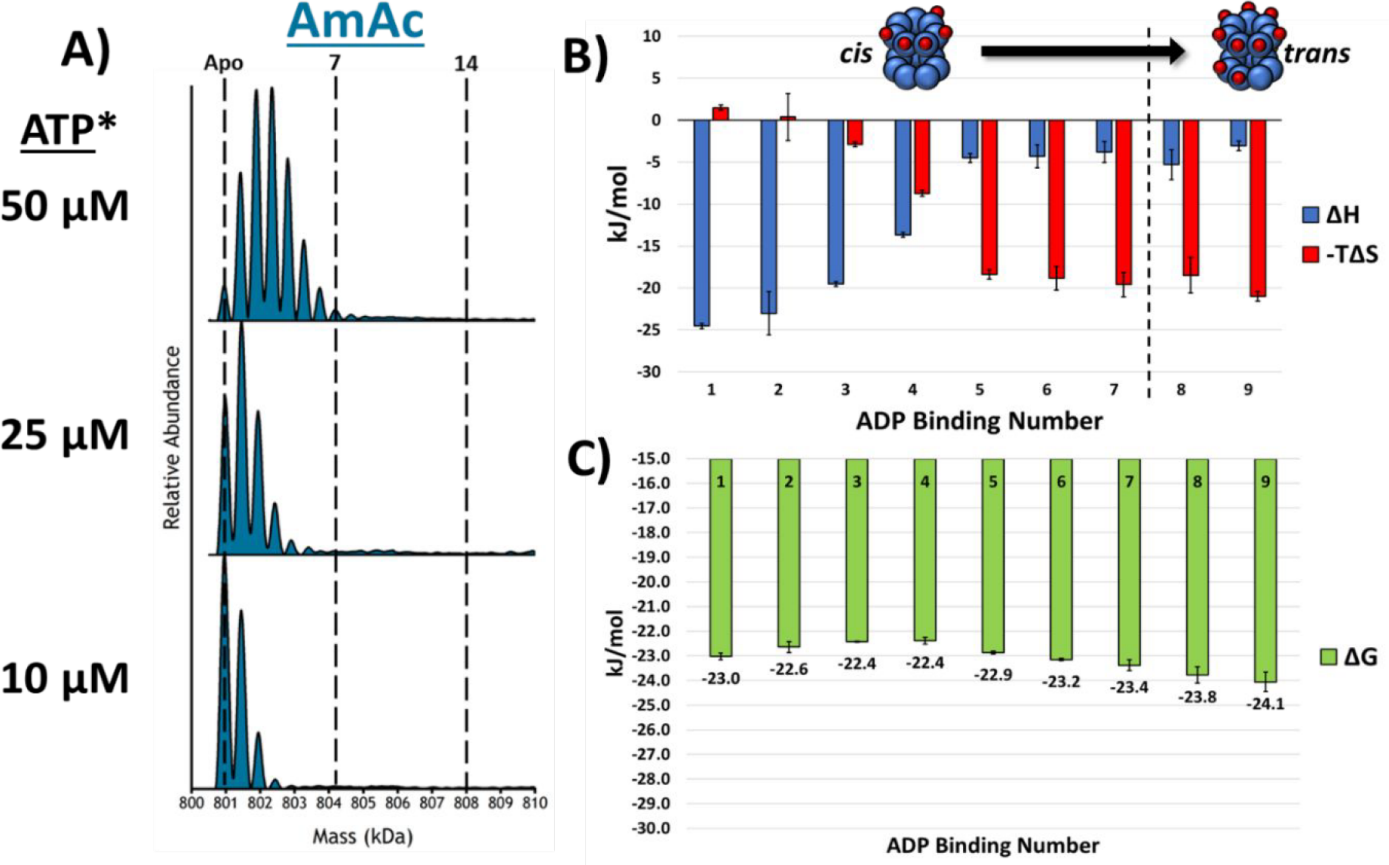
(A) Stacked deconvoluted spectra showing that as the ADP concentration is increased, the binding of ADP does not show any cooperativity. (B) Bar chart showing the Δ*H* and −*T*Δ*S* contributions at 25 °C for each of the ADP binding reactions. The enthalpy and entropy show a singular transition from GroEL–ADP_4–5_ and are overall much less dynamic than those for the binding of ATP in EDDA. (C) Bar chart displaying the Gibbs free energy measurements for the ADP binding reactions at 25 °C. EEC is more heavily present in the ADP data set, as the Gibbs free energy varies by less than 2 kJ/mol. All error bars are standard deviations of at least three replicates. *ATP was added to the solution, but only ADP binding was observed.

Ammonium acetate is a commonly used native MS buffer, but monovalent ions (NH_4_^+^, K^+^, and Rb^+^) are known to catalyze GroEL ATP hydrolysis.^51,52^ Measurements of individual nucleotide binding reactions using vT-nESI are used here in an effort to understand whether ammonium ions are directly or indirectly linked to the effects of ATP binding and subsequent hydrolysis. In AmAc solutions we only observe GroEL–ADP products (*vide infra*). Figure 4A contains the deconvoluted mass spectra for ADP-concentration-dependent binding to GroEL. When this is compared to Figure 2, it is clear that GroEL binds fewer ADPs than ATPs (in EDDA), and moreover, the changes in Δ*H*, −*T*Δ*S*, and Δ*G* are also significantly different from those for GroEL–ATP in EDDA. Figure 4B,C contains thermodynamic data for GroEL–ADP_*n*_ binding at 25 °C; the thermodynamic constants were only calculated up to GroEL–ADP_9_ because of poor fits for the van’t Hoff plots for GroEL–ADP_10–14_ (see Figure S3). The differences for enthalpy and entropy for GroEL–ADP_4–5_ compared to those for GroEL–ADP_1–3_ and GroEL–ADP_5–9_ and the Δ*G* values show that the ADP binding affinities follow a different trend from that of GroEL–ATP in EDDA solutions.

**Figure 5:**
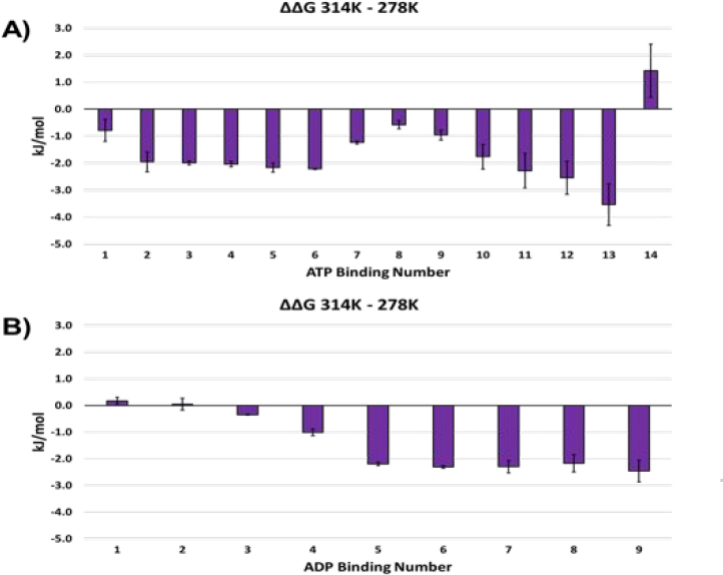
Histograms of ΔΔ*G* for GroEL–ATP (EDDA) and GroEL–ADP (AmAc). The more negative ΔΔ*G* correlates with more favorable nucleotide binding.

**Figure 6:**
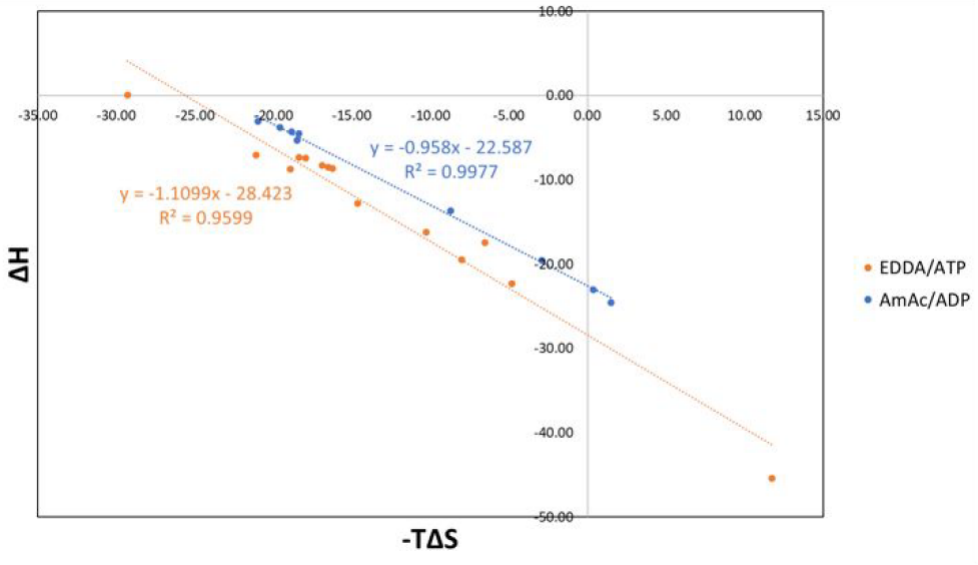
Plot comparing EECs for GroEL–ATP (EDDA) and GroEL–ADP (AmAc). The slope of the fitted line is close to unity, where perfect EEC should have a slope of 1.

The results for GroEL–ADP in Figure 3A–C are consistent with the known effects of monovalent ions (K^+^, NH_4_^+^, and Rb^+^) on hydrolysis of ATP to ADP by GroEL tetradecamer.^48−50^ Similar allosteric transitions (TT ⇔ TR ⇔ RR) described for GroEL in EDDA solution should be operative in AmAc solution, albeit at lower rates than that observed for solutions containing K^+^ ions. The differences in the observed EEC for GroEL–ADP (Figure 4C) suggest that there are minimal entropic barriers accompanying ADP binding, at least for the first and second binding reactions; however, addition of three to nine ADPs is subject to increasing entropic effects similar to those observed for GroEL–ATP in EDDA solutions, which suggests that binding of the first three or four ADPs does not perturb the conformation of the GroEL complex; however, subsequent addition of ADP induces entropically favored conformational changes. It is interesting that there are differences in the ΔΔ*G* plots shown in Figure 5, but these differences are fairly small and should not be overinterpreted. It should also be noted that the compensation plots (Figure 6, showing Δ*Hvs* −*T*Δ*S*) for EDDA/ATP and AmAc/ADP are quite similar and have similar slopes. The similar slopes of the compensation plots suggest that both GroEL–ATP and GroEL–ADP responding in a similar way to the addition of ATP and ADP, *viz.*, the compensation is a reflection of the overall protein dynamic conformational changes, structure of the complex, and the bound water.^16^

The nested cooperativity model proposes that a positive (MWC-like) transition governs ATP binding within each GroEL ring, while a negative (KNF-like) transition resists the simultaneous binding of ATP to both rings.^38,43^ In the absence of nucleotide, both GroEL rings primarily populate their low-affinity states (TT). ATP binding to one ring then favors an MWC-like (TT ⇔ TR) transition as the ring fills, while negative cooperativity inhibits ATP binding to the second ring and involves a KNF-like (TR ⇔ RR) transition. Because vT-ESI-MS permits the temperature dependence of each nucleotide binding event to be examined, the free energy balance between these key conformational and binding equilibria can be measured, revealing new mechanistic insight into this essential allosteric mechanism.

The affinities (intrinsic *K*_a_) for GroEL–ATP binding in EDDA are temperature-dependent (Figure 1), showing a trend of increasing binding affinity (Δ*G*) with increasing temperature. There are, however, notable differences in *K*_a_ at 23 °C relative to 41 °C, but even larger increases at 5 °C. Moreover, these changes are most pronounced for the binding of the first seven ATPs compared to the last seven ATPs; see comparisons of temperature-dependent Δ*G* (Figure 2) and ΔΔ*G***(**Figure 5). There are two limiting ways in which these results could be interpreted. First, ATP binding to a T-state ring might have a strong thermal dependence, so that the T-state affinity is much higher at low temperatures than at high temperatures and low occupancy rings (*e.g.*, *n* = 1 or 2) therefore bind ATP more strongly at lower temperatures. Alternatively, the first allosteric equilibrium constant (for the TT ⇔ TR transition; traditionally given the symbol “L”) might possess a very strong thermal dependence. In other words, if L for the first MWC-like transition is shifted to a much lower set point at low temperature, then the ring could flip from T to R at a far lower fractional saturation (*e.g.*, at *n* = 1 or 2) than can occur at higher temperature. Of course, the observed free energy pattern could also result from a combination of these two effects. Similar trends for the *K*_a_ values were noted by Dyachenko *et al.*(38) at 25 °C, which were interpreted as strongly supporting the nested cooperativity model.^42^ Our data, while also consistent with the main predictions of this model, also suggest that the equilibria between TT, TR, and RR states are strongly temperature-dependent. At the same time, a marked increase in the *K*_a_ (5 °C) for formation of GroEL–ATP_*n*_ (*n* > 7) suggests that binding of the seventh ATP at low temperature is linked to an additional conformational change. This shift, which cannot be detected at higher temperature, appears to be distinct from the T ⇒ R transition that occurs at lower ring occupancy. It is tempting to speculate that this conformational change is associated with the subsequent shifts of an ATP-bound R-state ring (*e.g.*, R2, R3), which are thought to be necessary for GroES binding and initiation of protein folding^33,43,54−56^ and become sufficiently stabilized at low temperature to be observed.

Similar arguments can be used to explain the enthalpy-favored binding of seven, eight, and nine ATPs to the second ring. At this point, the first ring should be fully in the R state, and the KNF-like transition to the RR state should be primed. The reduced ΔΔ*G* observed for this transition could be reasonably interpreted to indicate that the negative KNF-like transition also has a strong thermal dependence that is relaxed at lower temperature, so that the filling of both rings with ATP becomes far less unfavorable as the temperature drops. In fact, the observed favorable enthalpy and entropy for binding of seven, eight, and nine ATPs suggests that the free energy penalty normally associated with the TR ⇒ RR transition may have actually flipped to favor filling of the second ring with ATP.

## Conclusion

Variable-temperature electrospray ionization native mass spectrometry (vT-ESI-nMS) is a relatively new approach that complements isothermal titration calorimetry for studies of the effects of solution temperature on protein–ligand interactions (Δ*G*, Δ*H*, and *T*Δ*S*). Furthermore, the vT-ESI-nMS approach affords capabilities for determination of these quantities for individual binding reactions. Here we demonstrate the utility of vT-ESI-nMS through dissection of the thermodynamics for ATP binding to the GroEL tetradecamer one nucleotide at a time. By measuring these reactions as a function of temperature, we are able to determine equilibrium association constants (*K*_a_) followed by van’t Hoff analysis for determinations of Δ*G*, Δ*H*, and −*T*Δ*S* for each of the 14 ATP binding reactions. The observed differences for ATP binding to the *cis* and *trans* rings of the GroEL tetradecamer complex exemplify nested cooperativity in which intraring binding is concerted (MWC) and inter-ring communication is sequential (KNF). These mechanistic differences are also revealed by differences in entropy, as *cis*-ring ATP binding is highly entropically favored whereas EEC for *trans*-ring binding is variable. It is especially interesting to note the difference in EEC for nucleotide binding in EDDA *versus* AmAc solutions, where ADP binding in AmAc solution is largely enthalpy-favored for the *cis* ring but entropy-favored for the *trans* ring. In both cases, the free energy changes for binding to a given ring are quite small but variable between the two rings. Differences for enthalpy and entropy associated with binding of the last (14th) ATP in EDDA and the last two (13th and 14th) in AmAc should not go unnoticed, as these are highly enthalpically driven in EDDA but entropically driven in AmAc. The entropy components are indicative of structural changes of complexes and/or hydrating water network, whereas changes in the enthalpy (and the overall free energy in the AmAc buffer) reflect changes in the stability of the complex. Lastly, the primary source of the differences observed for EDDA and AmAc solutions is ATP hydrolysis to ADP.

These thermodynamic data show complex patterns for enthalpy–entropy compensation (EEC) for GroEL–ATP and GroEL–ADP. It is especially noteworthy that different thermodynamic mechanisms are observed for ATP binding to the *cis* and *trans* rings, *viz.*, formation of GroEL–ATP_1–7_ and GroEL–ATP_8–14_, and the observed EEC also shows significant dependences on the native ESI-MS buffers, ethylenediammonium diacetate (EDDA) and ammonium acetate (AmAc), *viz.*, differences arise owing to hydrolysis of ATP to ADP in AmAc buffer, which is not observed in EDDA buffer since NH_4_^+^ as the surrogate of K^+^ is necessary for GroEL ATPase activity. The MS data also support the existence of significant cooperative binding of ATP in EDDA solutions, which is also enhanced in AmAc solutions for hydrolysis-deficient GroEL^D398A^ mutant. The synergistic effects of monovalent cations in binding of ATP and ATPase activity in GroEL remain an interesting aspect that necessitates further investigation to elucidate their role in manipulating the conformational landscape of the chaperonin complexes. Studies employing ion mobility mass spectrometry, which is being used to access the extent of conformational changes accompanying ATP binding and subsequent ATP hydrolysis, are currently underway.

## Methods

### Sample Preparation

GroEL tetradecamer and GroEL^D398A^ tetradecamer were expressed and purified by the Rye research lab at the Texas A&M Department of Biochemistry and Biophysics. Aliquots of the GroEL samples were stored at −80 °C in a Tris buffer. Aliquots were buffer-exchanged into AmAc or EDDA (obtained from Sigma-Aldrich) using BioRad biospin P-6 size exclusion (6000 Da cutoff) columns to remove unwanted salt contamination. MgAc_2_ and Na-ATP were obtained from Sigma-Aldrich, and fresh solutions were prepared prior to each experiment.

### Experimental

Data were collected on a Thermo Q Exactive ultrahigh mass range (UHMR) mass spectrometer. Constituents for each sample were mixed immediately prior to analysis. For the thermodynamic analysis of GroEL–ATP binding, the solution conditions were 1 mM MgAc_2_, 200 mM EDDA, 500 nM GroEL (14-mer), and varying concentrations of ATP. The vT-nESI device was used to modulate the temperature of the solution; more information pertaining to operation of the device can be found in previous work.^13^ Solution temperatures used for this study were 5 to 41 °C; above 41 °C degradation products of the GroEL complex begin to become observable. The resolution setting was maintained at 25000 with five microscans; the injection time was 200 ms; the capillary temperature was 150 °C; the trap gas pressure was set to 7.0 (N_2_); the desolvation voltage (in-source trapping)^51^ was set to −200 V; and the HCD energy was set to 200 V (the latter two energy parameters only apply to EDDA buffer conditions). Care was taken to ensure that the gas-phase stability of the GroEL complex was retained; no monomer loss was observed for the energy setting listed previously. Also, loss of nucleotide ligands is highly unlikely due to reports that nucleotide binding in the gas phase is irreversible. This was tested experimentally at high collision energies, in which loss of ATP-bound monomers of GroEL was detected, signifying that the complex will dissociate prior to loss of nucleotide ligands in the gas phase (data not shown). The acquisition time for each spectrum was set to 1 min. Under these conditions, the ATP-bound states of GroEL were nearly baseline-resolved in most circumstances (Figures S4 and S5). Thirteen solution temperatures (every 3 °C from 5 to 41 °C) at eight ATP concentrations (0, 1, 5, 10, 15, 25, 35, and 50 μM) were analyzed in *n* = 3 trials. Each trial entailed the preparation of new solutions for buffers, GroEL, MgAc_2_, and ATP solutions.

### Data Processing

Each spectrum was deconvoluted using UniDec^57^ and incorporated the four most abundant ATP distributions (see Table S1 for assignment statistics). Baseline reduction was avoided in the UniDec software to avoid biasing the data. The area of each peak to be integrated was determined by extrapolating the local minimum between peaks down to baseline to serve as the limit of integration. The resulting relative abundances were used in a sequential binding model to fit dissociation constant (*K*_d_) values.^10,58^ The reciprocal of *K*_d_ is equal to *K*_a_; the natural logarithms of the *K*_a_ values were plotted against inverse temperature (in K) for van’t Hoff analysis (Figures 2D and S3). The slope of the fit line was used to calculate Δ*H* and the *y*-intercept was used to calculate Δ*S* in accordance with the equation
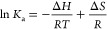
Using Δ*G* = Δ*H* – *T*Δ*S*, the Gibbs free energies were calculated in units of kJ/mol.

## Supporting Information Available

More thermodynamic data along with peak assignment data, examples of raw spectra, and van’t Hoff plots (PDF)
