# Supplementary material for: Dissecting the Thermodynamics of ATP Binding to GroEL One Nucleotide at a Time
Source: ACS Cent Sci. 2023 Feb 20;9(3):466–75. doi: 10.1021/acscentsci.2c01065 (PMC10037461; doi:10.1021/acscentsci.2c01065)
Supplement: Supplementary file 1 — oc2c01065_si_001.pdf [file oc2c01065_si_001.pdf]

Supplemental Information

Dissecting the Thermodynamics of ATP Binding to GroEL One Nucleotide at a Time

Thomas Walker,[1] He Mirabel Sun,[1] Tiffany Gunnels,[3] Vicki Wysocki,[2] Arthur Laganowsky,[1] Hays Rye,[3] and David Russell[1*]

[1] Department of Chemistry, Texas A&M University, College Station, TX 77843
[2] Department of Chemistry and Biochemistry, The Ohio State University, Columbus, OH 43210
[3] Department of Biochemistry & Biophysics, Texas A&M University, College Station, TX 77843

*Corresponding author, email: russell@chem.tamu.edu

Table of Contents **Page**

**Table S1**	**ATP assignment statistics**	**S2**
**Figure S1**	**Mole fraction vs ATP concentration plots**	**S2**
**Figure S2**	**Van't Hoff plots for GroEL-ATP$_n$ binding**	**S3**
**Figure S3**	**Change in ΔG in response to change in temperature**	**S4**
**Figure S4**	**Van't Hoff plots for GroEL-ADP$_n$ binding**	**S5**
**Figure S5**	**Representative spectra of GroEL-ATP$_n$ binding**	**S6**
**Figure S6**	**Representative spectra of GroEL-ADP$_n$ binding**	**S7**

Table S1. ATP assignment statistics. Average mass shift and error associated with the assignment of each [ATP + Mg^{2+}] (theoretical mass is ~531 Da) binding.

ATP #	Average Mass Shift (Da)	Standard Deviation (±Da)	% Error
1	548.6	18.4	3.31%
2	551.5	20.0	3.87%
3	536.5	3.9	1.04%
4	538.7	2.4	1.44%
5	539.7	16.0	1.63%
6	522.5	26.6	1.61%
7	531.4	6.6	0.07%
8	539.9	14.5	1.67%
9	542.4	3.0	2.15%
10	539.1	8.0	1.53%
11	519.1	26.4	2.25%
12	523.7	8.3	1.37%
13	527.4	6.9	0.67%
14	551.6	17.6	3.89%

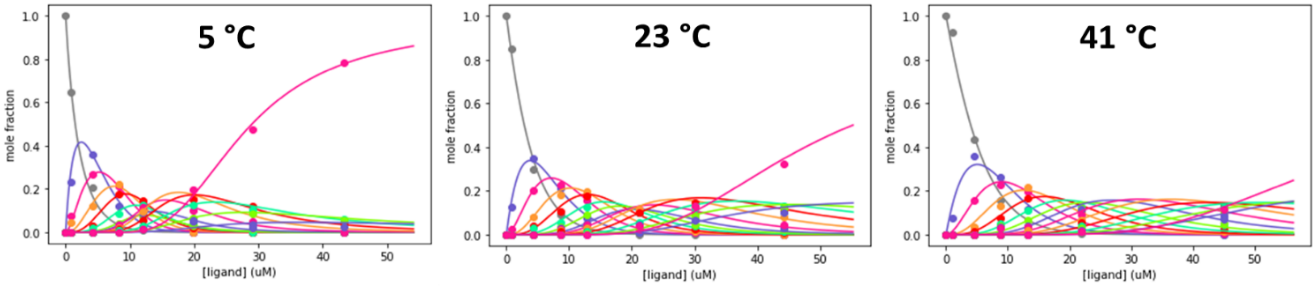

Figure S1. Mole fraction vs ATP concentration plots are used to calculate the K_a values. Three temperatures are shown and the n=14 binding event (pink line) is shown to decrease in relative abundance as temperature is increased.

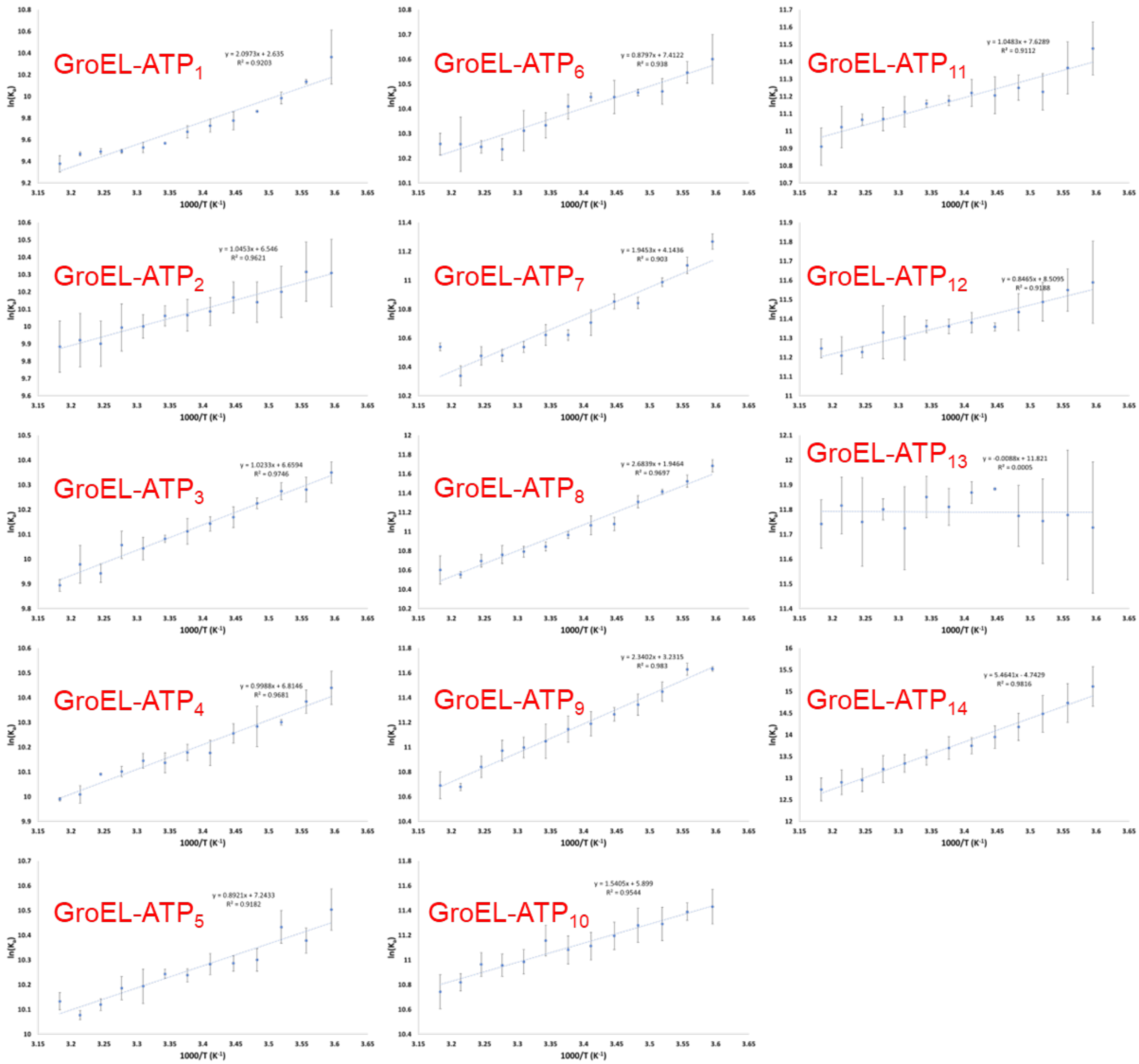

Figure S2. Van't Hoff plots for GroEL-ATPn binding in 200 mM EDDA (K_a vs 1000/T (K)).

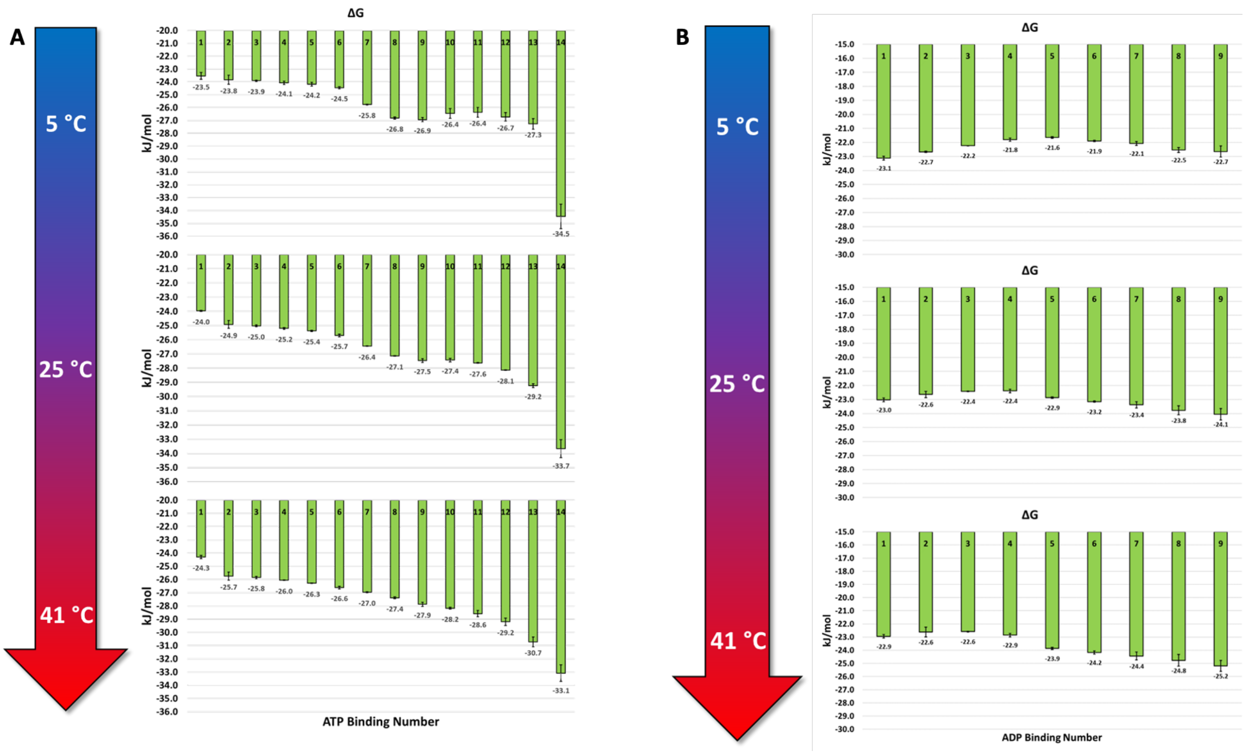

Figure S3. **Changes in ΔG at various temperatures. (A)** Stacked plots displaying the change in ΔG in response to change in temperature for GroEL-ATPn in EDDA. As the solution temperature increases, the bimodality observed for the sequential ATP binding becomes diminished; **(B)** Stacked plots displaying the Change in ΔG in response to change in temperature for **GroEL-ADPn in AmAc.**

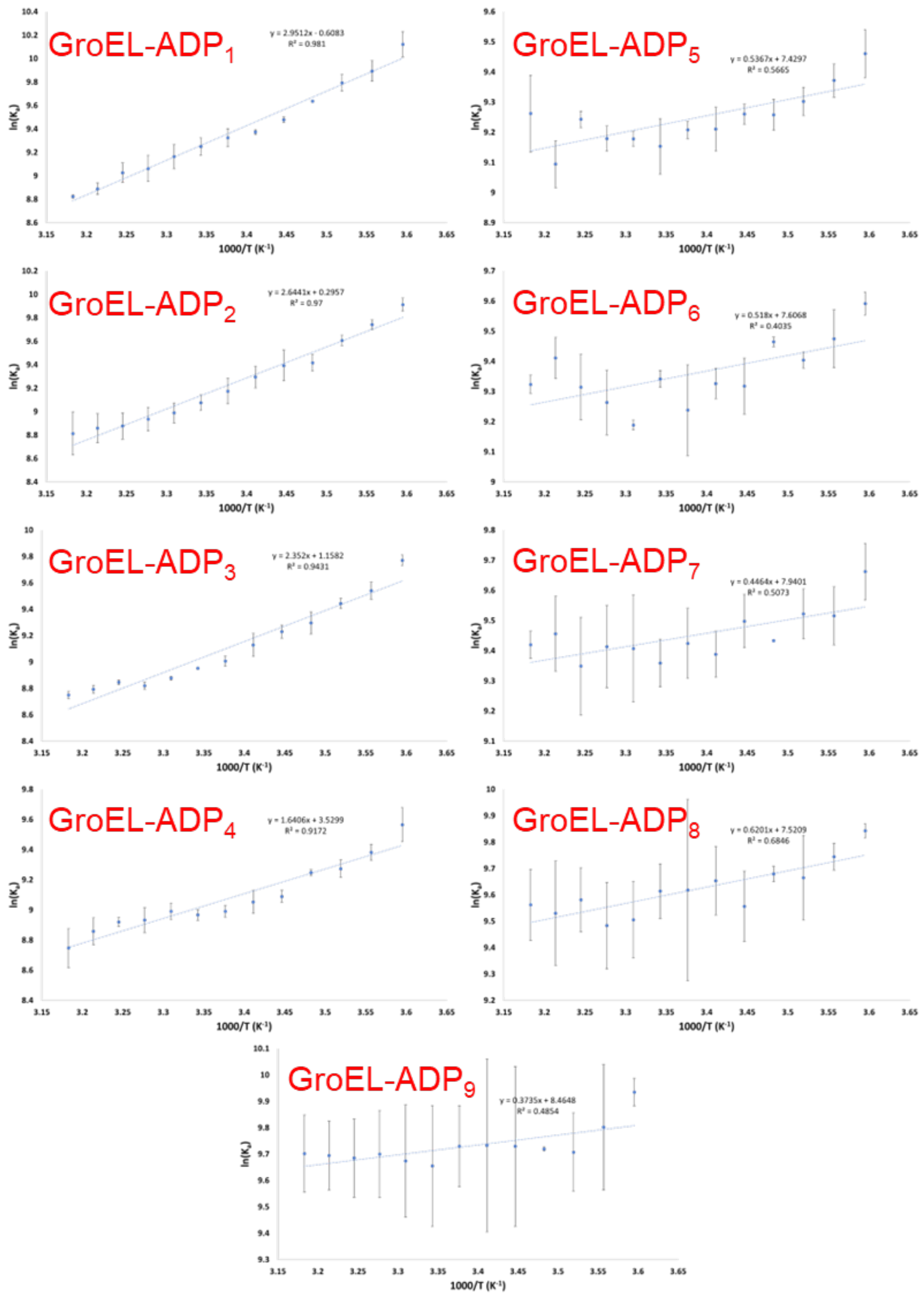

Figure S4. Van't Hoff plots for GroEL-ADP$_n$ binding in 200 mM AmAc (K_a vs 1000/T (K)). **Note:** ATP was added to the solution but only ADP binding was observed.

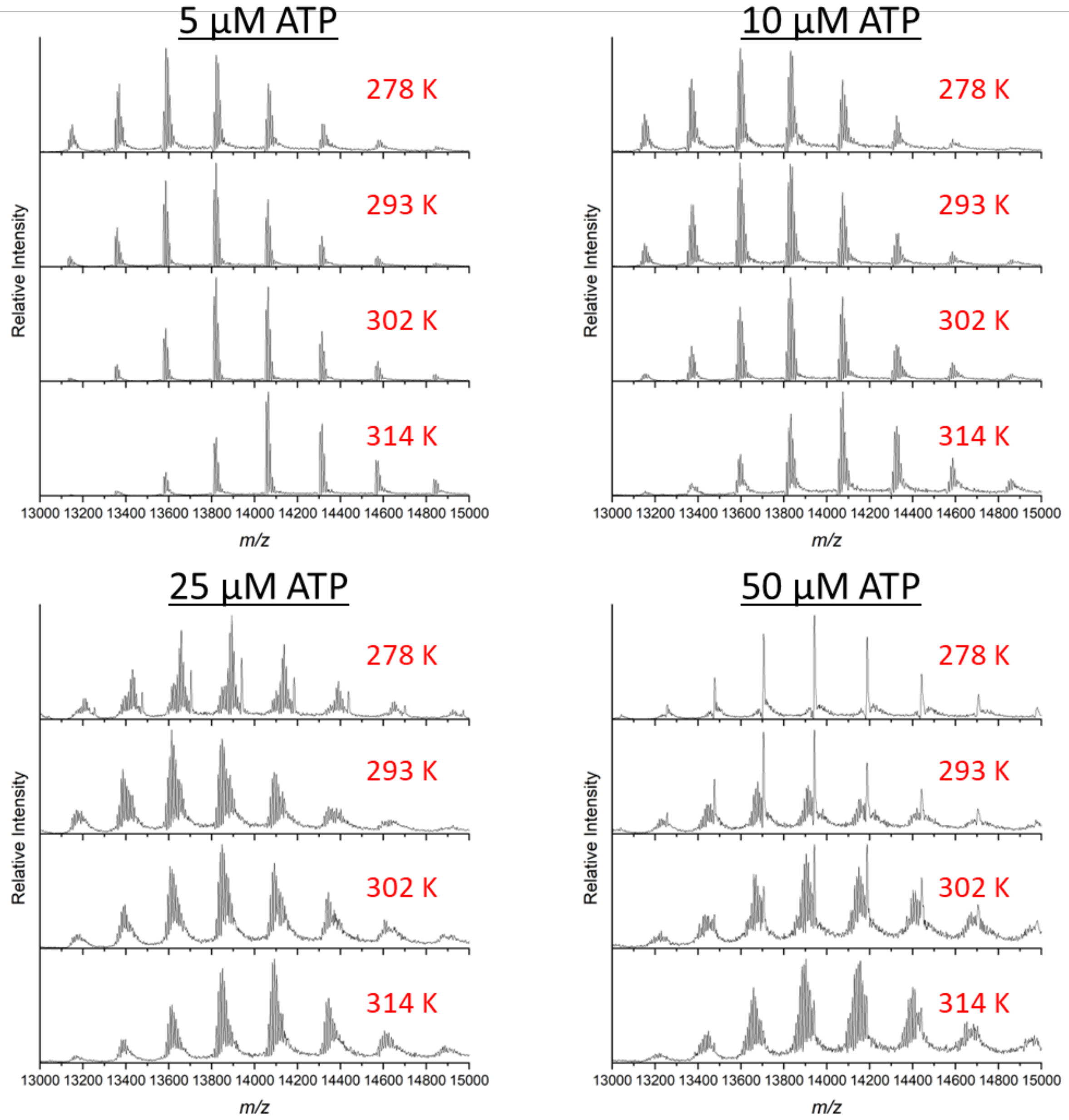

Figure S5. Representative spectra of GroEL-ATPn binding at various temperatures and ATP concentrations. Overall, ATP binding signals were well resolved as demonstrated above. Solution conditions are 500 nM GroEL, 200 mM EDDA, 1 mM $MgAc_2$, and binding various concentrations of ATP.

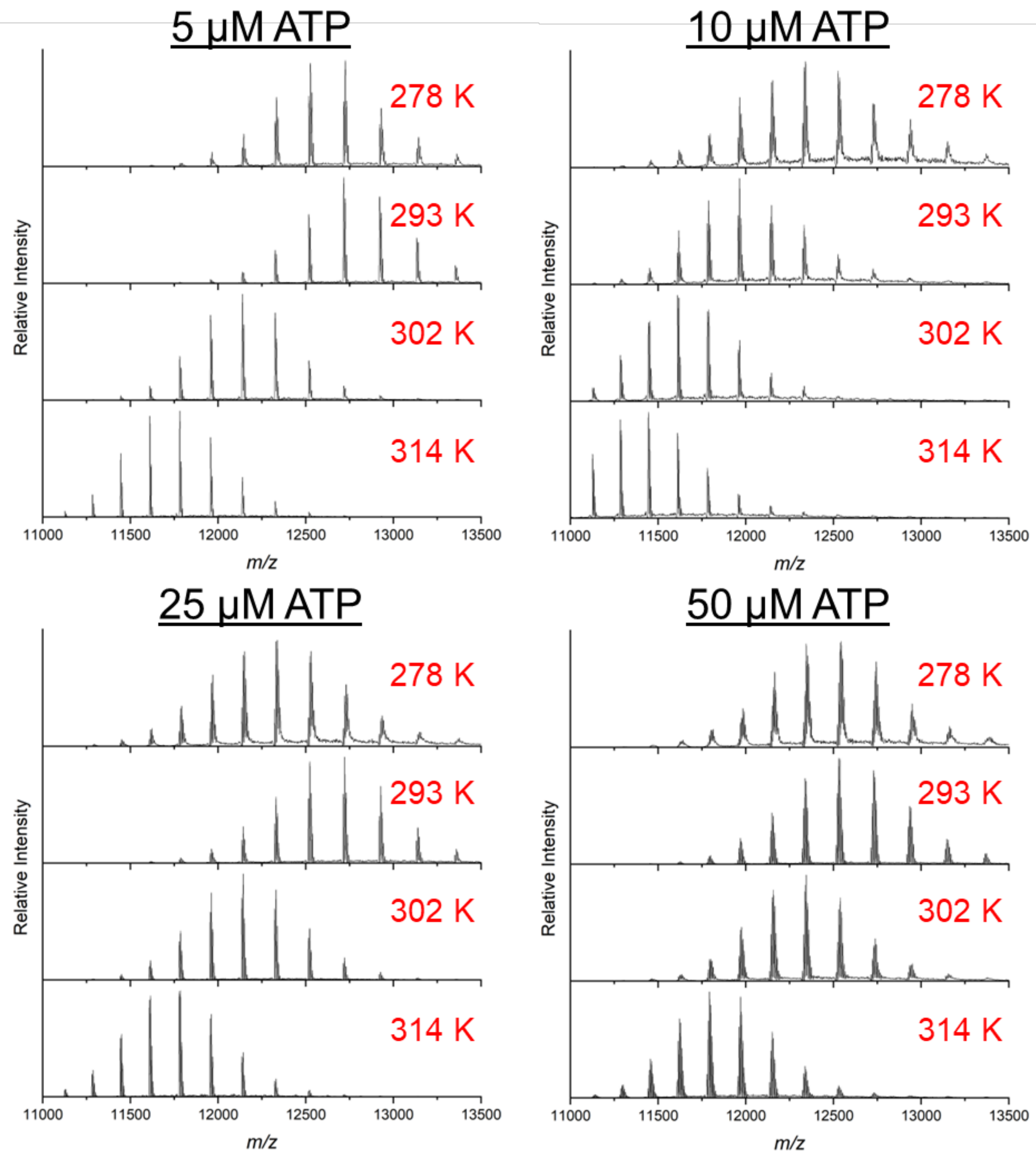

Figure S6. Representative spectra of GroEL-ADPn binding at various temperatures and ATP concentrations. Overall, ATP binding signals were well resolved as demonstrated above. Solution conditions are 500 nM GroEL, 200 mM AmAc, 1 mM $MgAc_2$, and various concentrations of ATP. Note: ATP was added to the solution by only ADP binding was observed.
